# Complete Genome Sequence of the First Isolate of Hibiscus Latent Singapore Virus Detected in Japan

**DOI:** 10.1128/genomeA.00054-18

**Published:** 2018-02-15

**Authors:** Tetsuya Yoshida, Yugo Kitazawa, Yutaro Neriya, Naoi Hosoe, Yuji Fujimoto, Yuka Hagiwara-Komoda, Kensaku Maejima, Yasuyuki Yamaji, Shigetou Namba

**Affiliations:** a Department of Agricultural and Environmental Biology, Graduate School of Agricultural and Life Sciences, The University of Tokyo, Tokyo, Japan; b Department of Agrobiology and Bioresources, School of Agriculture, Utsunomiya University, Tochigi, Japan; c Department of Sustainable Agriculture, College of Agriculture, Food and Environment Sciences, Rakuno Gakuen University, Hokkaido, Japan

## Abstract

The complete genome sequence of the first Japanese isolate of hibiscus latent Singapore virus (HLSV-J) was determined. The genomes of HLSV-J and a reported isolate from Singapore had only 86.7% nucleotide identity, while the encoded proteins shared amino acid identities of more than 95%.

## GENOME ANNOUNCEMENT

Members of the genus *Tobamovirus* in the family *Virgaviridae* are rod-shaped plant viruses with a single-stranded positive-sense RNA genome comprising at least four open reading frames (ORFs) and 5′ and 3′ untranslated regions (UTRs). A hibiscus-infecting tobamovirus, hibiscus latent Singapore virus (HLSV), was discovered in Singapore (HLSV-SIN; GenBank accession number AF395898) (1) and subsequently found in Australia (GenBank accession number AY664875), Taiwan (GenBank accession numbers AY546633 to AY546636) (2), and Italy (3). The complete nucleotide sequence of HLSV-SIN (4) and some partial sequences of other isolates have been determined.

In 2013, we collected hibiscus leaves with mild mosaic in Japan. Using electron microscopy, tobamovirus-like particles were observed in crude extracts of the leaves. The virus particles were purified, and viral RNA was extracted as described previously (5, 6). The extracted RNA was randomly amplified using a TransPlex complete whole-transcriptome amplification kit (Sigma-Aldrich, USA). The amplified products were cloned into the pCR-2.1-TOPO vector (Invitrogen, USA) and sequenced. These sequences showed 89 to 92% identities with partial genomic sequences of HLSV isolates, and we designated this isolate the HLSV Japanese isolate (HLSV-J). Two overlapping fragments in the genomic region, excluding the 5′ and 3′ ends, were amplified by reverse transcription-PCR and sequenced directly. To determine the complete sequence of HLSV-J, the 5′ and 3′ terminal fragments were amplified using a GeneRacer kit (Invitrogen) and a small-RNA cloning kit (TaKaRa, Japan), respectively. Both terminal fragments amplified were cloned into the pCR-Blunt II-TOPO vector (Invitrogen) and sequenced.

We determined the complete nucleotide sequence of HLSV-J. The HLSV-J genome was predicted to contain four ORFs encoding 186-kDa and 128-kDa replication proteins, a movement protein (MP), and a coat protein (CP). Sequence alignment with HLSV isolates using the MUSCLE algorithm in the program SDT (7) showed the following nucleotide/amino acid identities: 86.2%/97.1% (186-kDa protein), 85.3%/96.8% (128-kDa protein), 86.3 to 86.6%/97.5 to 98.2% (MP), and 89.2%/95.7 to 96.3% (CP). The 5′ UTR of HLSV-J and HLSV-SIN shared 96.6% identity. The 3′ UTR of HLSV-J contained a unique internal poly(A) region (42 to 100 nucleotides), as did HLSV-SIN (77 to 96 nucleotides) instead of the pseudoknot structure typical of tobamoviruses. Including the 42-nucleotide poly(A) tract, the HLSV-J genome was 6,441 nucleotides long. Excluding the poly(A) tract, the sequence identity of the 3′ UTR between HLSV-J and HLSV-SIN was 98.1%, and the complete genome identity between them was 86.7%. Based on the species demarcation criteria for the genus *Tobamovirus*, viruses that share less than 90% overall nucleotide sequence identity are considered distinct species (8). In this case, although the overall nucleotide sequence identity between HLSV-J and HLSV-SIN was less than 90%, the amino acid sequences of their ORFs and the nucleotide sequences of the UTRs shared more than 95% identity. Therefore, it seems appropriate to consider HLSV-J to be an isolate of HLSV and not a new *Tobamovirus* species. To our knowledge, this is the first report of HLSV in Japan.

### Accession number(s).

The genome sequence of HLSV-J possessing the 42-nucleotide poly(A) tract was deposited in the DNA Data Bank of Japan under the accession number LC127213.
